# Evolving management of pyogenic liver abscess: A narrative review of laparoscopic and open drainage approaches with contextual insights from Pakistan

**DOI:** 10.12669/pjms.42.(11AASC).15674

**Published:** 2026-04

**Authors:** Sidra Batool, Mohomed Ammaar Ghouse, Syed Mashhad Raza Shah

**Affiliations:** 1 Sidra Batool, MBBS. Dow Medical College, Dow University of Health Sciences (DUHS), Karachi, Pakistan; 2 Mohomed Ammaar Ghouse, MBBS. Dow Medical College, Dow University of Health Sciences (DUHS), Karachi, Pakistan; 3 Syed Mashhad Raza Shah, MBBS. Dow Medical College, Dow University of Health Sciences (DUHS), Karachi, Pakistan

**Keywords:** Hepatic Abscess Management, Hepatic Abscess Management in Pakistan, Laparoscopic Drainage, Open Surgical Drainage, Pyogenic Liver Abscess in Pakistan, Pyogenic Liver Abscess

## Abstract

**Objectives:**

Pyogenic liver abscess remains a high-risk condition, where clinical management has improved with modern advancements. In our study, we have attempted to compare the outcomes of laparoscopic and open surgical drainage as surgical management options, with contextual insights from Pakistan, where local studies remain scarce.

**Methodology:**

Articles were obtained from PubMed, Cochrane, ScienceDirect and Google Scholar from 2000 to 2025 using keywords such as “pyogenic liver abscess,” “laparoscopic drainage,” and “hepatic abscess management.” Outcomes, including mortality, morbidity, recurrence, hospital stay, and recovery time, were analyzed. Four comparative studies formed the core evidence and were supported by other reports.

**Results:**

We found that both laparoscopic and open drainage are effective in reducing mortality and recurrence rates. However, laparoscopic drainage was associated with lower morbidity, shorter hospital stays, and faster recovery duration. Recurrence had a variable result across studies, with favorable outcomes for the laparoscopic approach. Limitations faced include the limited availability of comparative studies, which were mostly single-centre, thereby not providing an overall picture of the situation in Pakistan.

**Conclusion:**

Our takeaway is that laparoscopic drainage is safer, more effective, and preferable where feasible in comparison to open-surgical treatment for surgical management. We also highlighted that access to laparoscopic drainage in lower- and middle-income areas should be enhanced through resource integration and capacity building for better healthcare. There is also a need to conduct further research, including more comparative studies, randomized controlled trials, and long-term cohorts, to gain a comprehensive comparative understanding of the various surgical approaches and potential future approaches.

## INTRODUCTION

Liver abscess is a potentially life-threatening consequence of microorganisms invading and subsequently multiplying, causing a suppurated cavity within the healthy liver parenchyma.^1^

The microorganisms that cause this vary based on the geographical presence but can be mainly separated into bacterial (pyogenic), protozoal (amoebic), mixed (pyogenic superinfection of parasitic abscess), or, more rarely, fungal causes. The liver abscess burden in the Western Hemisphere includes the pyogenic causes, mainly whereas a greater burden of liver abscess due to amoebic causes, such as from *Entamoeba histolytica*, in the South Asian, African, and Southeast Asian settings exists due to the prevalence of the protozoa and the generally poor hygiene conditions as compared to the developed countries.^2^

While percutaneous drainage continues to be the gold standard treatment for pyogenic liver abscesses, surgical drainages of open and laparoscopy are indicated in patients who fail to respond to percutaneous drainage and to antibiotics, patients with hepatic abscesses larger than five cm, multilocular hepatic abscesses, ruptured hepatic abscesses, requiring surgical intervention for underlying causes such as biliary tract disease, having lesions that are inaccessible by the percutaneous route or in areas where interventional radiology is not readily available.^1^ There are limited local studies over the past five years comparing the efficacy and efficiency of open and laparoscopic drainage when surgical intervention becomes necessary.

## METHODOLOGY

This narrative review was conducted through a comprehensive literature search using international databases, including PubMed, Science Direct, Cochrane and Google Scholar. The search covered studies published between 2000 and 2025, focusing on the management of pyogenic liver abscess (PLA) through different drainage techniques. Keywords such as “pyogenic liver abscess,” “laparoscopic drainage,” “antimicrobial therapy,” and “hepatic abscess management” were utilized.

### Exclusion criteria:

It included non-English articles, non-pyogenic liver abscess and studies focusing exclusively on percutaneous drainage outcomes.

### Inclusion criteria:

Relevant peer-reviewed articles, clinical trials, case reports, and review papers were included, prioritizing the latest studies. Data were extracted, analyzed, and synthesized qualitatively to identify current trends, report treatment approaches, compare efficacy of drainage techniques, and highlight advancements in minimally invasive procedures with a regional perspective from Pakistan.

## PYOGENIC LIVER ABSCESS OVERVIEW

### Pathogenesis and Etiology of the Infection:

Infection can spread hematogenously, either via the portal vein from abdominal sources such as infections or pylephlebitis or, less commonly, from distant sites such as ENT (ear, nose, and throat) or the oral cavity.

Issues within the biliary systems, often involving gallstones, obstructed biliary ducts, contiguous spread, or ascending cholangitis, also create an additional pathway for infection. The abscess may also alternatively arise from existing lesions or anomalies of the liver, such as superinfected metastases, necrosis within a primary tumor, biliary or hydatid cysts, or conditions such as Caroli disease and biliary strictures.^1^

*E. coli, K. pneumoniae* and Streptococcus have been observed as the most common etiologies in identification. Enterococci and *S. viridans* are commonly identified in polymicrobial abscesses. Causative micro-organisms differ based on the etiology; that is, penetrating trauma wound may reveal *S. aureus* and *S. pyogenes*, biliary disease presence may isolate *Enterococci* and enteric gram-negative organisms, and a portal seeding etiology may reveal Coliforms and anaerobes.

### Epidemiology and Clinical Presentation:

Liver abscesses occur in about 2.3 per 100,000 cases and are significantly higher in lower socioeconomic groups and developing countries. Males are more frequently affected by liver abscesses than women.^3^ Mortality rates range from 5-10% in North America and Europe to 3-30% worldwide.

Risks that are associated with the development of liver abscess and the increase of morbidity and mortality of patients include increasing age, male sex, presence of diabetes mellitus, liver cirrhosis, previous liver procedures, continuous use of proton pump inhibitors, and immunocompromised state.^4^

The patient may present with multiple nonspecific symptoms, including fever, nausea, vomiting, and weight loss. Jaundice is a common physical manifestation of Pyogenic Liver Abscess (PLA). Other physical signs include right upper-quadrant abdominal pain and hepatomegaly. Delayed presentation and the higher disease burden in developing countries increase the likelihood of large, complex abscesses, which are more likely to require surgical drainage.^5^

### Diagnostic Evaluation:

Laboratory testing for diagnostic evaluation in PLA reveals elevated levels of globulin, Alkaline Phosphatase (ALP), Aspartate aminotransferase (AST), alanine aminotransferase (ALT) and total bilirubin, along with anemia, leukocytosis, and prolonged prothrombin time.^5^

Imaging is central to diagnosis and differentiation. CT scan is the most accurate imaging modality over ultrasound for locating hepatic abscesses, number, and identifying the probable source; with contrast-enhanced CT preferred. PLA typically appears as a hypodense area that doesn’t take up the injected IV contrast. A thin, enhancing rim is a classic sign that is described as a “ring sign.” The presence of internal gas is unique and is pathognomonic. Imaging must also be utilized to detect underlying causes such as biliary disease and pylephlebitis or other infections, while suspected biliary or hepatic vein involvement can be further investigated using MRI.^1^,^5^ Microbiological evaluations include **bile cultures,** that detect microorganisms in up to 95% of cases, and **blood cultures**, which have a detection rate of approximately 52%.

### Classification of Pyogenic Liver Abscess:

Pyogenic liver abscesses can be clinically differentiated into two categories, namely, the simple and complex categories. Simple liver abscesses are small, solitary in nature, and unilocular. They are less than 5 cm in diameter, have a thin wall, have minimal septations, and can be managed with percutaneous drainage along with a course of antibiotics, especially in young patients.

Complex liver abscesses are multilocular and exceed five cm in diameter. They are thick-walled, numerous, and are generally managed by drainage of the abscess, which is performed either via open or laparoscopic surgery.^4^,^6^ Complex liver abscess forms the keystone for this narrative review. It is imperative that the abscess be diagnosed and differentiated into one of the two categories to optimize the treatment plan.

**Fig.1:**
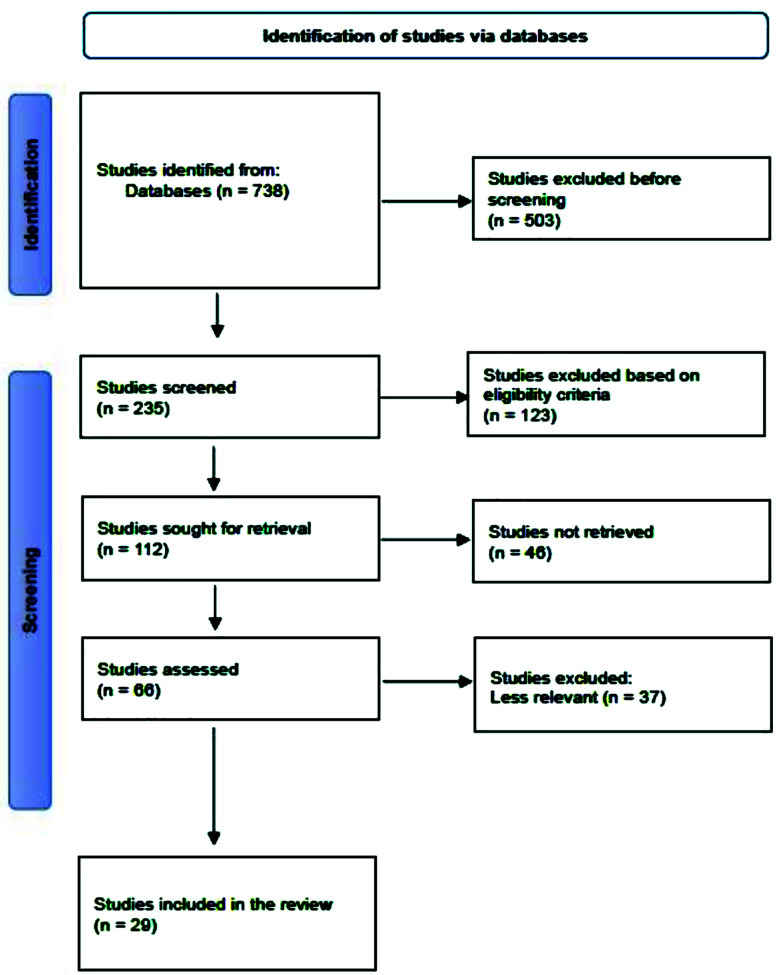
Overview of screening process.

### Treatment Options:

The treatment options for PLA are based on the etiology of the abscess as mentioned above. It is necessary that an Amoebic liver abscess be ruled out before commencement of the treatment.

### Pharmacological Treatment:

Our literature review indicates that the primary approach to treating PLA is antimicrobial therapy, which may or may not be combined with drainage, depending on the abscess size. It is recommended that, as soon as PLA is suspected, broad-spectrum empirical antibiotic treatment be promptly initiated after collection of culture samples.^7^

The primary purpose of the initial antibiotic regimen is to eradicate the underlying bacterial infection within the abscess cavity and bloodstream, to prevent bacteremia, recurrence, or systemic sepsis. Therefore, the empirical broad-spectrum plan should cover typical gram-positive bacilli, notably K. pneumoniae and E. coli, which Pakistani cohorts report as the leading causes of PLA, as well as streptococcal species and anaerobes.^8^,^9^

Since microbes develop antibiotic resistance over time, specific medicinal treatment plans should be tailored as per the patient’s need. In this regard, local resistance patterns also play a critical role in drug selection.^8^ An optimal antibiotic course is imperative as a first line treatment for pyogenic liver abscesses, before exploring other options.

### Historical Shift of Drainage Methods:

To comprehend the efficacy of drainage techniques for pyogenic liver abscess, it is essential to consider the historical evolution from conventional open surgical drainage to modern procedures. Before antimicrobial therapy and advancements in radiology, open surgery through laparotomy to drain pus was the cornerstone of hepatic abscess management. However, the treatment approach has shifted from routine open surgical drainage to minimally invasive, image-guided methods like percutaneous drainage that now achieve mortality rates below 5%, which has been possible with the advent of diagnostictherapeutic radiology and broad-spectrum antimicrobial therapy; current cohorts report no deaths after percutaneous drainage and antibiotics alone.^10^

Contemporary guidelines recommend percutaneous drainage as the first-line intervention for abscesses larger than five cm, whereas smaller lesions can often be treated with antibiotics only.^11^,^12^ Intermittent needle aspiration also has comparable efficacy to PLA and may be considered first-line due to its simplicity, patient comfort, and lower cost.^13^

Surgical drainage is still reserved for a subset of patients, including those with failure of percutaneous catheter drainage, multiloculated or septated lesions, large or complex abscesses, rupture, peritonitis, or concomitant biliary disease.^10^,^14^ However, treatment failure is relatively common with percutaneous drainage in large abscesses compared to surgical management.

### Surgical Treatment Options:

Surgical interventions are of two types: Open drainage, involving laparotomic access to the PLA, and the modern innovation, Laparoscopic drainage (LD), via laparoscopic access to the PLA.

### Open Surgical Drainage:

Open drainage is carried out in a laparotomic fashion, wherein a right subcostal or a midline incision is made, depending on the abscess location. Intraoperative ultrasound is used to determine the abscess site and size. Post identification, the abscess is then located, drained, and the fibrous mesh within the septa is broken down to ensure complete evacuation of the abscess material. Hemostasis is secured, and a latex drainage tube is left in the cavity to ensure postoperative drainage.^6^

The available literature on OS in PLA management is mainly based on studies conducted before 2010, and information on the role of open drainage in PLA management is scarce. OS is rarely utilized in modern healthcare practice and opted for only in specific complex cases.^12^

However, historically, open surgery served as both a therapeutic and diagnostic method in treating PLA, with a mortality rate peaking at 65% before the 1970s when it was the mainstay treatment.^15^ With improved imaging techniques and effective antimicrobial treatment, however, there is a declining trend of reliance, as indicated in retrospective research based on 64 patients suffering from PLA, where it was indicated for only 12% of the patients.^16^

### Laparoscopic Surgical Drainage:

Laparoscopic drainage is performed under general anesthesia, where after establishing the pneumoperitoneum, a 10 mm trocar is inserted for the laparoscope. An additional five mm port is inserted, and an alternative 10 mm port can be used for intraoperative ultrasound. Adhesions between the liver, bowel, and the anterior abdominal wall are released to expose the abscess. The site and extent of the abscess are identified via the ultrasound, and the abscess is aspirated using a suction catheter. 6

In modern practice, laparoscopic surgery is considered a safer and more favorable option in scenarios where percutaneous drainage or antibiotic therapy do not yield satisfactory clinical results, as it allows drainage of pus without requiring laparotomy, with the additional perk of exploration of the common bile duct. It is increasingly preferred over the invasive open resection unless surgery is inevitable, with success rates similar to open procedures.^12^,^17^

### Future Prospects:

In the current age of artificial intelligence, rapid next-generation technological advancements, such as robotic surgery, augmented reality, and AI-assisted decision-making in healthcare, are constantly changing the way morbid diseases are managed.

Technologies like augmented and mixed reality (AR/MR) project a three-dimensional computer model of the liver onto the surgeon’s view, helping locate the abscess and guide the drainage; where early case series show registration errors of about 5-9 mm, making the approach feasible for minimally invasive liver work.^18^

Combining laparoscopic drainage with AR/MR, three-dimensional navigation, or robotic assistance could make the procedure safer, reduce operative time, and expand its use to more complex abscesses. As these tools become widespread and clinical data emerges, the future of liverabscess treatment and hepatobiliary surgery in general is likely to become “digital guided,” laying the foundations of prospective futuristic healthcare techniques.

## DISCUSSION

### Aftermath: Clinical Outcomes and Implications of Laparoscopic versus Open Drainage in Pyogenic Liver Abscess:

With the evolving clinical management of pyogenic liver abscess (PLA), it is essential for clinicians to assess the comparative outcomes of available surgical options. This review compares two key intervention techniques, laparoscopic drainage (LD) and open surgical drainage (OD), drawing primarily from Aslam et al.^3^ and supported by additional prospective and retrospective studies as well as case reports. Reviewing the available evidence, mortality and recurrence rates were found similar for both approaches; however, LD showed lower postoperative complications, shorter hospital stays, and faster recovery. These insights may assist clinicians to select the most effective invasive management strategy for PLA, supporting informed and patient-centered decision-making.

### Morbidity:

There is a consistent evidence across studies in favor of laparoscopic drainage resulting in lesser rate of morbidity which is in-line findings reported by Aslam et al. except for one study where the difference was reported as insignificant.^19^ This lesser morbidity rate poses LD as a preferred option to be considered for immunocompromised and elderly patients where recovery rate is inherently lowered.

However, the recurrence rate has been shown to be variable across studies, with Aslam et al. 2023 indicating a comparable rate of recurrence. This finding is validated by other studies where recurrence, if occurred, was resolved by percutaneous drainage.^3^,^6^,^19^,^20^ However, some single-center case series attribute laparoscopic drainage with a higher prospective of leading towards a recurrent event, with a meta-analysis pooling data from twenty years of study analysis accrediting LD with a recurrence rate of 4.22%.^14^,^21^,^22^ The recurrence event is a concomitant effect of existing comorbidities, previous abdominal surgeries, and tumors.^23^

**Table-I:** Comparative outcomes of Laparoscopic (LD) and Open surgical drainage (OS) in Pyogenic Liver Abscess

Author (Year)	Study Design	Sample size OD/LS	Clinical outcomes: Morbidity	Clinical outcomes: Recurrence	Clinical outcomes: Recovery duration	Clinical outcomes: Mortality
Aslam Rz Shah FO, Shah MN, Abdullah M	Comparative	30/30	Lower morbidity for LD	Similar recurrence rate for both LD and OS.	Shorter hospital stay for LD	No mortality reported in both.
Mogahed MM, Zytoon AA, Eysa B, Manaa M, Abdellatif W.	Combined retrospective and comparative	26/22	No reported morbidity for LD; indicated morbidity in OS.	One recurrence case in both treatments.	Shorter hospital stay for LD.	No mortality reported in both managements.
Tu JF, Huang XF, Hu RY, You HY, Zheng XF, Jiang FZ.	Comparative	18/13	Higher morbidity for OS group.	No recurrence reported for LS; single recurrence case for OS.	Rapid recovery for LD group with earlier oral feeding commencement.	None.
Ansari PA, Mishra NK, Gupta SK..	Prospective, Comparative	30/30	Significantly lower post-operative complications for LD	Reduced recurrence rate for LD.	Rapid recovery for LD group with earlier oral feeding and routine activities commencement.	Not assessed.

The wound infection frequency was found to be higher in OS, which may be associated with the technique’s requirement of larger incision size and exposure to intra-abdominal contents, as opposed to the precise drainage granted by the laparoscopic method due to magnified visualization.^20^ A robust assessment of safety profiles of both methods, however, is indicated to draw a more definitive conclusion regarding the risks associated with each technique.

### Mortality:

Mortality remains low for both therapeutic methods, to the extent of being negligible amongst all of the examined studies^3^,^6^,^19^,^20^, and is only attributed to systemic high-risk factors such as the presence of co-morbidities, sepsis, or severe underlying pathology that is detected later.^24^

### Patient recovery duration:

The duration for patient recovery and resumption of daily routine is a crucial determinant of the efficacy of a clinical management method of a disease. It also has socioeconomic implications, as a prolonged hospital stay is a causative agent of enhanced hospital resource utilization and health-care system burden, in addition to hampering the patient’s quality of life and their contribution to society.^25^

Laparoscopic drainage, in this regard, has been promising, consistently indicating speedier recovery and reduced hospital stay duration in comparison to OS, which also underscores its long-term cost-effectiveness in the health sector.^19^ The post-operative feeding was also initiated earlier in the LD approach, indicative of its rapid resumption of regular bodily functions.^3^ The intraoperative time was also reportedly lower in LD; however, the difference was not large enough to consider it as a major feature and thus requires further investigation for the establishment of a definitive statement.^6^,^19^

### Limitations and Prospects for future:

Our review is the first of its kind to analyze head-to-head, the comparative outcomes of LD and OS across studies to establish associated risks and clinical features, and opens door for further thorough investigations of both therapeutic techniques for definitive comparative analysis.

We also attempted to emphasize the feasibility of the conduction of both management methods in a resource-restricted settings by opting our reference study from Pakistan, which has been not executed before. It highlights the investment that is required from public-health sector stake holders in the enhancement of improved therapeutic accessibility to patients and the gaps that exist in implementing and integrating them.

It should, although, be noted that the strength of our conclusions are limited due to paucity of comparative studies conducted, the single-institution settings and restricted sample size. The comparative studies had to be supported from case series and case reports, which marks a critical literature gap in the investigation of PLA management techniques. The conduction of randomized control trials and detailed long-term comparative studies that examine the lasting impacts of both methods on patient’s lifestyle, recurrence risks, post-operative complications, the difficulties and cost-effectiveness of both approaches are necessary to establish standardized criteria. There also needs to be analysis conducted for other patient outcomes which were not discussed, including but not limited to postoperative pain, patient tolerance, and cosmetic results.

It is also imperative to prioritize research that investigates contributing factors of recurrence rates and failure of both methods. The direction of investigation should be towards comprehension of influence of co-morbidities on patient outcomes in both managements and across diverse patient backgrounds.

## CONCLUSION

Both laparoscopic and open surgical techniques are effective techniques in terms of parameters of mortality, morbidity, and safety profiles, with similar results in patient outcomes except for morbidity and patient recovery duration, where LD promised more efficient outcomes, placing it as a preferred choice of therapeutic management before OS in cases of PLA not complicated by dense adhesions and concomitant surgery-requiring morbidities for producing similar results as open surgery. Thus, LD could be considered as a first-choice surgical management approach for cases of PLA not resolved by first-line treatments, for its benefits of rapid recovery time and lesser complications.

However, it should be noted that laparoscopic technique is reported with its limitation of tactile sensation that poses challenges for surgeons and underscores the conjunct use of ultrasonography.^26^ It should also be highlighted that the laparoscopy method, with its higher precision, requires a highly skilled, trained team of surgeons and equipment, both of which are highly constrained in lower-middle-income countries (LMICs) where liver abscess is prevalent. 14 This is relevant in the parent-study origin country, Pakistan, where although the prevalence of laparoscopic techniques is improving,^27^ yet is constrained from widespread integration into clinical practice. The major barriers are inadequate funding, lack of equipment, stakeholder dynamics, non-integration in training curricula, and decreased opportunity to practice. The most frequently reported cause is the lack of government support or health care policies to inculcate laparoscopic surgery into daily clinical practice. This also sheds light on a major public health issue in LMICs, where access to laparoscopy, if present, is restricted to major urban centers, intensifying health inequity and ease of access opportunities.^28^,^29^

### Author contributions:

**SB:** Conceptualization, Literature review, Writing original draft, Review and Editing.

**MAG:** Literature review, Writing original draft, Review and Editing.

**SMRS:** Literature review, Writing original draft.

All authors have read and approved the final version and are accountable for integrity of the study.
